# Primary Central Nervous System B-Cell Lymphoma Presenting with Fluctuating Basal Ganglia and Cortical Lesions

**DOI:** 10.5334/jbsr.4044

**Published:** 2025-08-18

**Authors:** Christophe Sonck, Jef Huyskens

**Affiliations:** 1 Vrije Universiteit Brussel Health campus, Laarbeeklaan 103, 1090 Jette, Belgium; 2 Radiology department, Azorg ziekenhuizen, Merestraat 80, 9300 Aalst, Belgium

**Keywords:** primary central nervous system lymphoma, primary central nervous system (CNS) tumors, basal ganglia, magnetic resonance imaging, FLAIR, B-cell lymphoma, brain neoplasms, stroke minics

## Abstract

A 68-year-old woman presented with progressive hemiparesis and dysphagia, initially presumed to have subacute stroke. Serial MRI revealed fluctuating FLAIR hyperintensities and enhancing lesions in the basal ganglia and cortex, ultimately leading to the diagnosis of high-grade primary CNS B-cell lymphoma. The case highlights the importance of integrating clinical evolution with imaging features in atypical neurological presentations.

*Teaching point:* In cases of fluctuating FLAIR hyperintensity and contrast-enhancing brain lesions, primary CNS lymphoma should be considered, even in the absence of typical imaging features.

## Introduction

Primary central nervous system lymphoma (PCNSL) is a rare but aggressive non-Hodgkin lymphoma confined to the brain, leptomeninges, spinal cord, or eyes. Diagnosis is often delayed due to nonspecific symptoms and imaging features that can mimic other pathologies, including stroke or infection. A case of PCNSL is described, presenting with atypical and fluctuating imaging findings, further emphasizing the diagnostic challenges and the importance of multidisciplinary evaluation [1, 2].

## Case Report

A 68-year-old female presented with progressive left hemiparesis, dysphagia, and leftward tongue deviation. Past medical history included presumed ischemic events. Neurological examination revealed 2/5 motor strength in the left upper extremity and bilateral hyporeflexia. Ophthalmologic evaluation showed left visual loss, attributed to cerebrovascular disease. Electromyography suggested a mixed central and peripheral neurogenic process.

**Figure 1:**
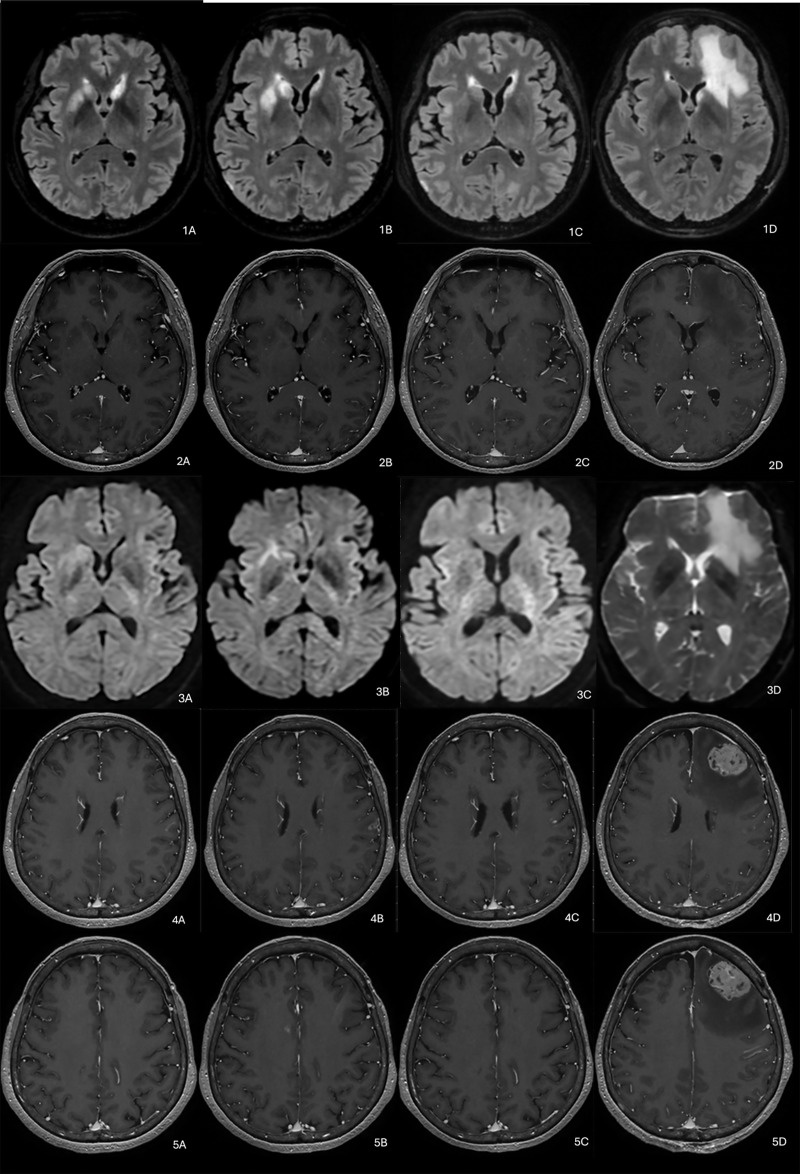

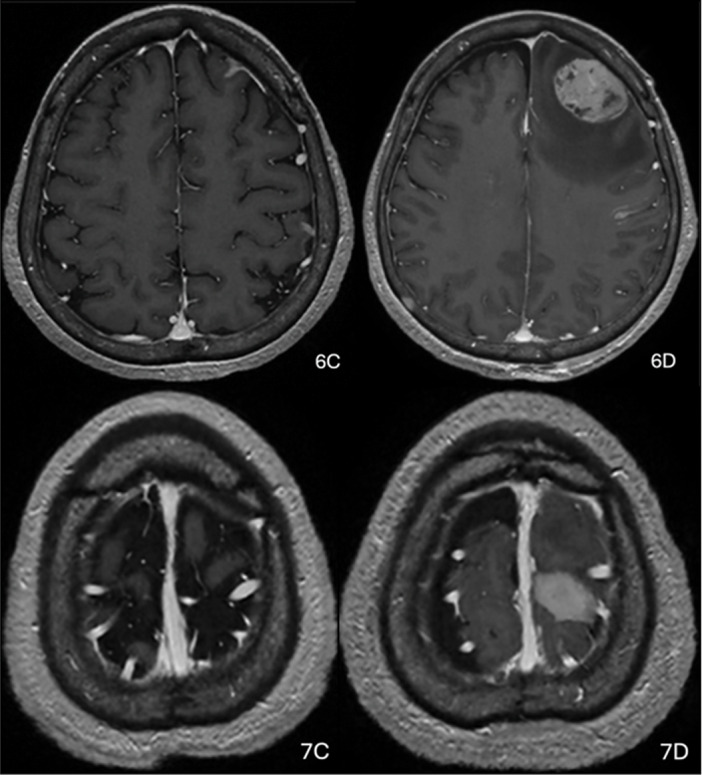
Axial brain MRI scans acquired at four different time points (“A,” “B,” “C,” and “D”). FLAIR sequences are labeled with “1,” while T1-weighted post-contrast images correspond to labels “2,” “4,” “5,” “6,” and “7.” Diffusion-weighted images (DWI), indicating potential diffusion restriction, are labeled with “3.”

Initial brain MRI (Figure 1, timepoint A), performed to investigate facial palsy, showed asymmetric FLAIR hyperintensity in the basal ganglia without contrast enhancement or diffusion restriction, inconsistent with acute infarction. Multiple small enhancing subcortical lesions were observed, prompting a broad differential diagnosis. One month later, MRI (Figure 1, timepoint B) showed progression of non-enhancing basal ganglia lesions and increasing volume of enhancing foci. Despite radiological progression, the patient clinically improved, supporting a presumptive diagnosis of subacute stroke.

Three months after the onset, the patient deteriorated abruptly. MRI (Figure 1, timepoint C) showed decreased basal ganglia FLAIR hyperintensity and fluctuating enhancing lesions, including a new extra-axial lesion in the left frontal region. These findings were considered atypical for ischemic stroke. A multidisciplinary discussion proposed differential diagnoses, including metabolic, infectious, or neoplastic processes [3, 4]. Given the lesion dynamics and clinical picture, primary CNS lymphoma became the leading hypothesis [1].

Two months later, MRI (Figure 1, timepoint D) showed further progression, with a dominant extra-axial lesion and a new high frontal lesion exerting mass effect. Biopsy of the dominant lesion confirmed high-grade B-cell lymphoma with high mitotic index and necrosis [3–5]. Chemotherapy was initiated.

## Discussion and Conclusion

PCNSL can exhibit highly variable imaging features, especially early in its course. Lesions may appear as non-enhancing FLAIR hyperintensities, which can evolve into enhancing masses. This case underscores that even in the absence of typical restricted diffusion or homogeneous enhancement, PCNSL must remain in the differential diagnosis when faced with fluctuating MRI findings [1–4].

Early multidisciplinary involvement and repeated imaging may be crucial for timely diagnosis. The dynamic nature of the lesions and clinical course ultimately pointed toward lymphoma. This case demonstrates the role of radiological follow-up in guiding tissue diagnosis and treatment planning in atypical CNS presentations [4, 5].
